# High-Profile VRU Detection on Resource-Constrained Hardware Using YOLOv3/v4 on BDD100K

**DOI:** 10.3390/jimaging6120142

**Published:** 2020-12-19

**Authors:** Vicent Ortiz Castelló, Ismael Salvador Igual, Omar del Tejo Catalá, Juan-Carlos Perez-Cortes

**Affiliations:** 1 Instituto Tecnológico de Informática (ITI), Universitat Politècnica de València, 46022 Valencia, Spain; vortiz@iti.es (V.O.C.); odeltejo@iti.es (O.d.T.C.); 2 Departamento de Informática de Sistemas y Computadores (DISCA), Universitat Politècnica de València, 46022 Valencia, Spain

**Keywords:** on-road detection, artificial intelligence, machine learning, convolutional neural networks, resource-constrained hardware, one-stage detectors, advanced driver-assistance systems, vulnerable road users

## Abstract

Vulnerable Road User (VRU) detection is a major application of object detection with the aim of helping reduce accidents in advanced driver-assistance systems and enabling the development of autonomous vehicles. Due to intrinsic complexity present in computer vision and to limitations in processing capacity and bandwidth, this task has not been completely solved nowadays. For these reasons, the well established YOLOv3 net and the new YOLOv4 one are assessed by training them on a huge, recent on-road image dataset (BDD100K), both for VRU and full on-road classes, with a great improvement in terms of detection quality when compared to their MS-COCO-trained generic correspondent models from the authors but with negligible costs in forward pass time. Additionally, some models were retrained when replacing the original Leaky ReLU convolutional activation functions from original YOLO implementation with two cutting-edge activation functions: the self-regularized non-monotonic function (MISH) and its self-gated counterpart (SWISH), with significant improvements with respect to the original activation function detection performance. Additionally, some trials were carried out including recent data augmentation techniques (mosaic and cutmix) and some grid size configurations, with cumulative improvements over the previous results, comprising different performance-throughput trade-offs.

## 1. Introduction

Object detection is one of the main tasks regarding Computer Vision, with the aim of detecting instances (objects) or continuous patterns (textures) in images or videos. Some principal cases of study are video surveillance, industrial inspection, face identification, and autonomous driving assistance, with particular challenges for each case.

Regarding the latter, much effort has been put into it lately, especially in the last decade, when it has been noticed that the fully autonomous vehicle (SAE 5) could become a reality in the foreseeable future. In the on-road context, different objects have to be confidently detected, mainly other vehicles, but with a special focus on the so-called Vulnerable Road Users (VRU), including pedestrians, cyclists, motorcyclists and road workers because they clearly constitute, with almost no exception, the weak party in any road traffic crash [1].

Just to report the magnitude of the problem, approximately 2 out of 3 fatalities involve VRU on urban road networks [2]. Besides this, the relatively reduced size when compared to other instances (e.g., vehicles) and the intrinsic variability in the VRU appearance (e.g., people in different clothes, poses and light conditions) makes it especially challenging to confidently detect them. Therefore, despite improvements in recent years, the VRU detection task still poses many difficulties that require further dedication on design, optimization, and assessment [3].

During the last two decades, many approaches have been developed with the aim of confidently detecting on-road objects, especially VRU. First, attempts included the use of traditional, computing extensive algorithms, such as AdaBoost and Cascade based detection structures, known as Viola–Jones (VJ) [4] or Histogram of Oriented Gradients plus Support Vector Machine structures (HOG+SVM) [5], usually leading to poor results.

Later, other models such as Deformable Part Model (DPM) [6], Integral Channel Features (ICF) [7], Locally Decorrelated Channel Features (LDCF) [8] or Fast Feature Pyramids (FFP) [9] were developed, with intermediate performances but depending on ad hoc, predefined features. Other approaches comprised adaptive neuro-fuzzy inference systems and feature descriptors such as Histogram of Oriented Gradients (HOG) and Local Binary Pattern (LBP) [10] or ad hoc preprocessing [11]. In addition, structured learning has been applied for training structured ensembles in order to address the pedestrian detection problem [12].

In the last decade, however, the dramatic increase in computing capacity that led to the development of Convolutional Neural Networks (CNN) made it possible to get much better object detectors, such as Region Based Rich Feature Hierarchies (R-CNN) [13], Faster R-CNN [14], Single Shot Multibox Detector (SSD) [15], and You Only Look Once versions (YOLO) [16,17,18,19].

Because of the high computational requirements of many CNN variants, some one-stage developments are focused on reducing it so as to run fast on mobile platforms (high throughput). In the past, these were considerably worse in terms of detection quality when compared to heavy, two-stage CNN algorithms [20], but nowadays the gap between them is being dramatically reduced [18].

For this reason, one-stage methods have become very common: they use a single pass to detect relevant objects of all aspect ratios at multiple scales in the image. They comprise lighter algorithms that are much more suitable for the available onboard hardware [19].

YOLO algorithms belong to the one-stage category and are used on on-road applications for object detection including instances such as pedestrians [21,22,23,24], vehicles [25], traffic flows [26], and non-helmeted motorcyclists [27].

In this context, many developments tend to rely on the MS-COCO dataset [28] for training, thus leading to well-balanced detectors for the 80 classes included. However, due to this high number of classes to be detected and their great context variability, the detection performance for these models is suboptimal when applied to on-road datasets, which have a reduced group of classes with a common context.

Because of that, we adapted both YOLOv3 and YOLOv4 models and trained them with Berkeley DeepDrive 100K (BDD100K), one of the most diverse and large automotive datasets captured on the street, including data from multiple cities. The dataset comprises day and night images, with different weather and adverse lighting conditions, with the focus on vehicles and VRU [29]. After that, we experimented with two new activation functions for the convolutional layers from YOLOv4, namely MISH [30] and SWISH [31], with promising results for this task.

As an additional development, we retrained the best of these algorithms with some recent data augmentation techniques, including mosaicand cutmix, with improvements from the previous results. In addition, we explored further the best algorithm performance for a wide range of grid configurations, with some specific findings due to this particular on-road detection task.

All this work is part of the European project PRYSTINE [32], which will strengthen and extend traditional core competencies of the European industry, research organizations, and universities in smart mobility and in particular in the electronic component and systems and cyber-physical systems domain. In particular, this development is part of the sensor fusion system to reduce uncertainty and to improve precision in the perception of the vehicle surroundings.

The paper is organized as follows: first, the detectors and the dataset used are presented. Then, the training settings and development are reported. Later, an ad-hoc training with a reduction of the number of categories is carried out for YOLOv3 and YOLOv4, leading to a significant increase detection performance. Additionally, YOLOv4 training processes are developed both for MISH and SWISH activation functions and some data augmentation techniques and grid sizes, with improvements in terms of detection performance. Finally, conclusions and future work on the topic are drawn.

## 2. Materials and Methods

Our experiments were aimed at obtaining the most accurate model for on-road detection suitable for a resource-constrained hardware platform, typically a mobile board (e.g., Nvidia Jetson TX2 or any other Nvidia Drive computer platforms). For this reason, due to our experience on the field and after thorough research among the latest developments regarding CNN for real-time object detection, we selected YOLOv3 and YOLOv4 as the most balanced net structures to train. These nets combine a relatively low computational load with an outstanding detection performance for the common 0.5 Intersection over Union (IoU) threshold due to some features such as batch-normalization and residual-connections, which are applicable to the majority of models, tasks, and datasets [18,19]. YOLOv4 variants were developed by improving and enhancing the YOLOv3 previous structures.

Furthermore, a bunch of different versions are available, with different input sizes. Once the most adequate algorithm versions were selected, a particular dataset or a combination of some had to be used to train the nets. Although generic-class databases, such as MS-COCO [28], are usually the starting point to develop general detection algorithms, we focused our effort on specific on-road databases, especially those including VRU.

**Figure 1:**
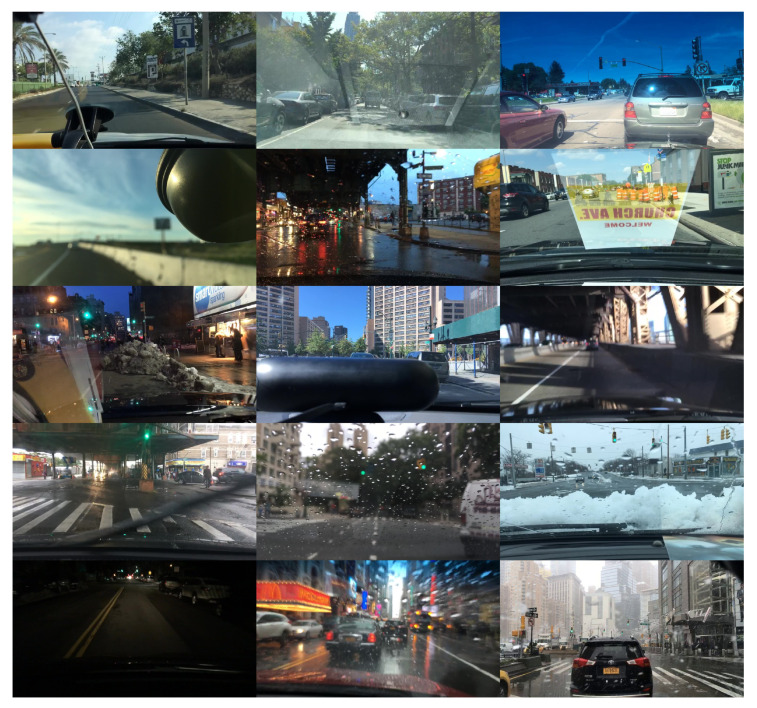
Image samples from BDD100K dataset, in which a great variability (occlusion, bad weather, glare, low light, low contrast, light reflection, blur...) can be noticed.

After extensive research on the literature of the last 10 years and according to the number of images, number of categories, dissemination and variety, we preselected some specific on-road databases, namely Caltech-USA [33], EuroCity Persons [34], and BDD100K [29]. However, by analyzing the particular class set, the image quality and the topicality from each one, we chose BDD100K to train the algorithms: a huge, complete dataset including different weather conditions, places and times of the day, and a wide range of light conditions, occlusion, and cropping. This semantic variety can be noticed in Figure 1.

Therefore, our aim was to train some YOLOv3 and YOLOv4 variants with BDD100K and to compare the performance of these models with their generic MS-COCO-trained counterparts when applied to the same on-road dataset and classes present in BDD100K. To achieve this, we used the original YOLOv4 repository [35] and semantically assigned some MS-COCO classes to the existing BDD100K ones. For every training process, we used a single Nvidia Tesla V100 GPU.

To assess the performance of each model, some metrics are especially useful. IOU is a commonly used basic metric that refers to the area overlap ratio of the obtained detection and the ground truth boxes and can be expressed as:

$$\mathrm{IoU}=\frac{A\cap B}{A\cup B}$$

Based on it, one of the main indicators used to compare different models is mAP50, which represents the Mean Average Precision at IoU = 0.5. In addition, Precision and Recall were assessed in the grid size analysis. All of these metrics are briefly explained below:

$$\mathrm{Precision}=\frac{\mathrm{TP}}{\mathrm{TP}+\mathrm{FP}}$$

$$\mathrm{Recall}=\frac{\mathrm{TP}}{\mathrm{TP}+\mathrm{FN}}$$

where TP, TN, and FN stand for true positives, true negatives, and false negatives, respectively. From these indicators, the AP (Average Precision) can be calculated as follows:

$$AP=\frac{1}{101}\sum_{i=0}^{100}\mathrm{Precision}\left(\mathrm{Recall}=\frac{i}{100}\right)$$

If IoU is selected as the overlap measure between the ground-truth and the detected bounding boxes, and a 0.5 lower threshold is defined to consider that a particular detection is carried out properly, the AP50 measure can be calculated for each class. Finally, mAP50 can be found as the weighted AP50 average for all classes.

Regarding the input–output relation, it consists of a net structure (predefined depending on the particular detector used, but modifiable to enhance its performance, as we did in the grid size analysis) and its weights (automatically obtained in the training process). Therefore, thanks to the deep learning approach, only the hyperparameters must be defined previously.

The global functioning of YOLO detectors [18,19] is as follows: they divide an image into grids at three different scales creating three grids of different sizes. Each cell of the grid is responsible for predicting up to three bounding boxes for objects whose center pixel is within the cell. Whereas the input of the algorithm consists of a single image (a frame), the output bounding box format is as follows:

$$\left(x,y,w,h,c,C_{1},C_{2},\ldots ,C_{n}\right)$$

in which *x* and *y* are the shifts relative to the top-left corner of the cell; *w* and *h* are width and height of the bounding box relative to some preselected anchor boxes; *c* is the confidence that YOLO estimates for the detection, and $C_{1},C_{2}\ldots C_{n}$ are the confidences of belonging to each class.

Finally, when the enhanced BDD100K trained YOLOv4 models were obtained, a retraining process was carried out replacing the original Leaky ReLU activation functions with two new, cutting-edge functions recently added called MISH and SWISH, with slight improvements from the original detections performances. Subsequent experiments used data augmentation techniques in the training process. Finally, a special postprocess (grid size) analysis was performed. Both approaches led to improved performances.

## 3. Experiments

In order to carry out our experiments, the first thing needed was to adapt the BDD100K dataset to the format that Darknet framework expects. In addition, after a semantic analysis of the classes present in BDD100K and the 80 MS-COCO classes, the assignment presented in Table 1 may be established among them.

Therefore, we considered these equivalences to compare the detection performance of both MS-COCO 80-class original model and ours, which was trained with BDD100K. In order to compare the detection performance of the newly BDD100K-trained models numerically, we assessed the mAP50 comparison between each of the BDD100K trained weights (10-class and VRU-class) and the official YOLOv3/v4 weights from the author. Of course, the assessment was carried out applying these algorithms to the same on-road dataset (that is, the BDD100K full test dataset).

### 3.1. YOLOv3-416 Training with BDD100K

The 416 × 416 input YOLOv3 structure was selected due to its balance between detection quality and throughput so as to meet the PRYSTINE project requirements in terms of computing capacity. First, we trained the YOLOv3-416 structure for three class sets: 10-class (full BDD100K classes), 7-class (main on-road classes) and VRU (person+rider, bike, car, and motor). Then, we tested them to compare their AP50 to the original 80-class model AP50 only for the corresponding classes. To perform this, we had to regenerate all the individual BDD100K label .txt ground-truth files with the classes of interest in each case only. These results allow us to compare between original and both BDD100K trained models, as the following results are obtained by analyzing the same test subset with the same algorithm and weights.

The training process was performed for the three class subsets (namely, the full 10-class from BDD100K, the main 7-class and the VRU-class models), with about 135k, 90k, and 80k iterations to achieve the best models, respectively, spending from 3.5 to 5 training days on a single V100 GPU. The detection performance in terms of AP50 for each model is shown in Table 2. Notice that the AP50 for the train class is near zero because the number of train instances in the BDD100K ground-truth is residual, thus leading to poor class learning. However, the detection of this type of vehicle is not foreseen in the PRYSTINE project context.

To establish a comparison, the original MS-COCO trained weights from the author were loaded in order to test this generic model when applied to an on-road dataset such as BDD100K. The results are shown in Table 3.

**Figure 2:**
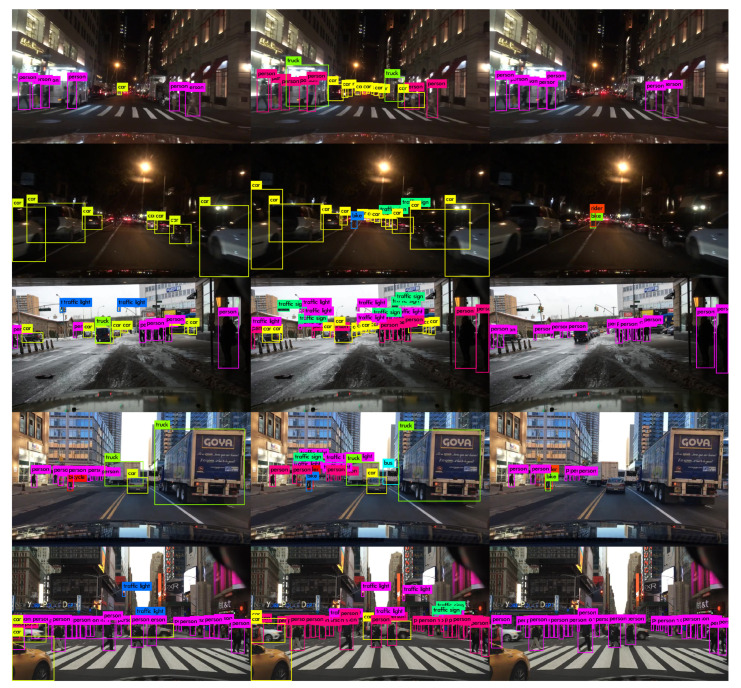
Some processed images from BDD100K test dataset with YOLOv3-416: original MS-COCO model from the author (**left** column), our BDD100K 10-class training (**central** column) and our BDD100K VRU-class training (**right** column).

The above data show that the increase in detection quality is noticeable for the three BDD100K trained models in terms of mAP50 when compared to the original 80-class MS-COCO model (45.15% vs. 26.65% for 10-class, 49.85% vs. 32.94% for 7-class and 42.10% vs. 29.02% for VRU-class). It can also be noticed that the 7-class trained model pushes forward the AP50 for these classes by about 1% on average, as it happens with the VRU-class model by about 2% on average, both with respect to the 10-class trained model. The performance of each YOLOv3 algorithm can be visually assessed in Figure 2, where some detected images from the test BDD100K subset are presented.

### 3.2. YOLOv4-416 Training with BDD100K

After a while, YOLOv4 model was released in different sizes. Therefore, the training process was replicated for the 7-class BDD100K subset, since it is the class subset of interest in the PRYSTINE project context for the recently launched YOLOv4-416 algorithm. From the original YOLOv4 paper, one can expect an increase in detection quality at the expense of a slight decrease in throughput, which we will check afterwards. As before, the training process was performed for the main 7-class from BDD100K, with about 90k iterations to achieve the optimal model and spending about 4.5 training days on a single V100 GPU. The detection performance in terms of AP50 for the trained model is shown in comparison with its YOLOv3-416 counterpart from the previous YOLOv3 section in Table 4.

**Figure 3:**
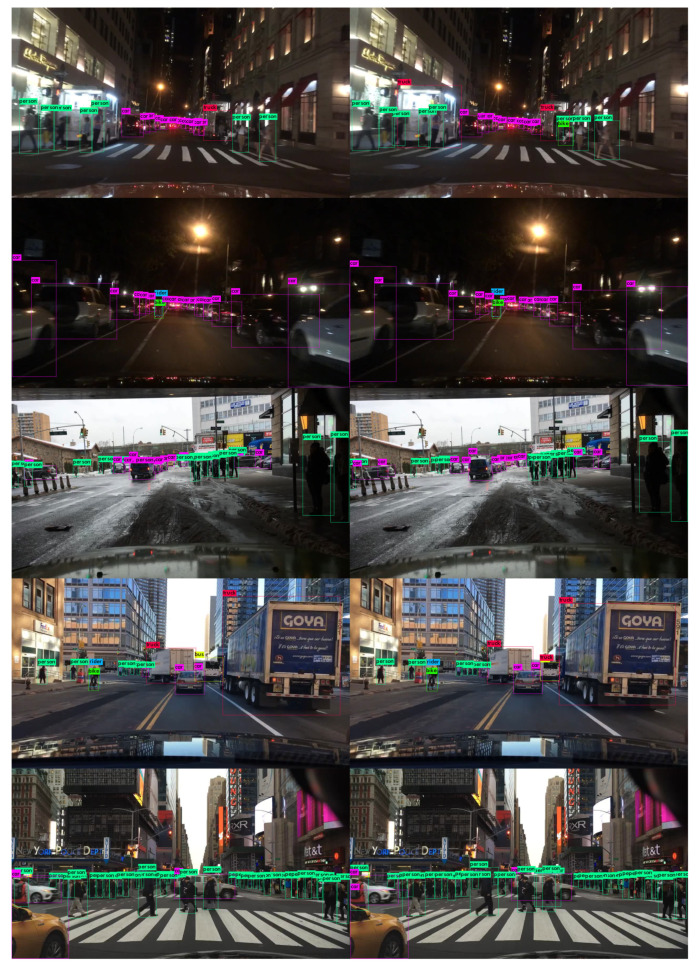
Some processed images from BDD100K test dataset with BDD100K trained models: YOLOv3-416 (**left** column) versus YOLOv4-416 (**right** column).

Again, the above data show that the increase in detection quality is noticeable for the YOLOv4 structure in comparison to the YOLOv3 one with the same 7-class subset. The new structure pushes forward the AP50 for these classes by about 2.4% on average. However, the forward-pass time on the 2 × Nvidia Jetson TX2 included in the final PRYSTINE demonstrator board (Nvidia Drive PX2 AutoChauffeur) also rose a bit, as it can be seen in Table 5. Additionally, the performance of both YOLO algorithms when detecting the 7-class set can be visually assessed in Figure 3, where some detected images from the test BDD100K subset are presented.

### 3.3. YOLOv4-416 Training with New Activation Functions: SWISH and MISH

Leaky ReLU [36] is one of the most common activation functions in current CNN which helped to solve the dying ReLU problem by introducing a slight slope in the negative side. Recently, two activation functions named SWISH [31] and MISH [30] have been proposed with reported improvements over Leaky ReLU. SWISH activation function is defined as

$$f\left(x\right)=\frac{x}{1+e^{-x}}$$

and MISH is defined as

$$f\left(x\right)=x*\tanh(\ln\left(1+e^{x}\right))$$

**Figure 4:**
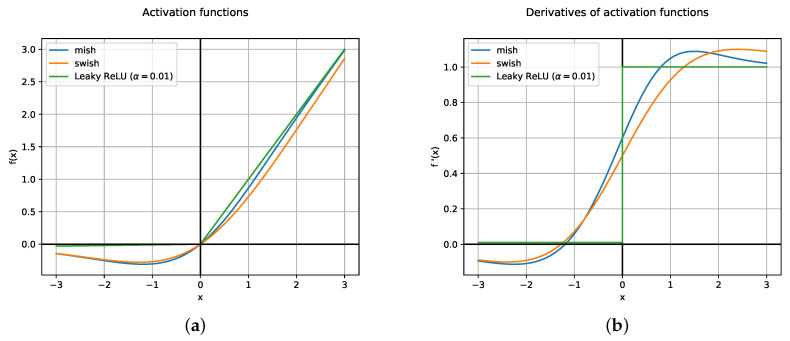
Leaky ReLU, MISH, and SWISH activation functions (**a**) and their derivatives (**b**).

SWISH activation function resembles ReLU, but it is smooth and non-monotonic in contrast to common activation functions. Just a simple replacement brings about an improvement in classification accuracy on ImageNet [37]. In addition, MISH activation function is inspired by the self gating property of SWISH (see Figure 4a,b).

Both functions present similar properties: being unbounded on the positive side avoids saturation which generally causes training to drastically slow down due to near-zero gradients. From the other side, the fact of being bounded in the negative side results in strong regularization effects and helps reduce overfitting. Moreover, in contrast to Leaky ReLU, SWISH and MISH are continuously differentiable, which is good for gradient based optimization. YOLOv4 makes use of MISH and reports an increase of 2.1% in mAP50 in the MS-COCO object detection task [19]. Particularly, MISH functions reside inside the backbone, which is known as CSP-Darknet53, but are not present in the rest of the architecture, where Leaky ReLU is used.

Given the performance achieved by SWISH and MISH functions on different object detection tasks, we propose to extend its use to the whole YOLO network. For this reason, Leaky ReLU functions in the neck section have been replaced by MISH functions (and the same for SWISH). These functions are located on the joint between the CSPDarknet53 backbone and the SPP module. In addition, Leaky ReLU activation functions inside PANet block have been substituted for MISH/SWISH functions.

**Figure 5:**
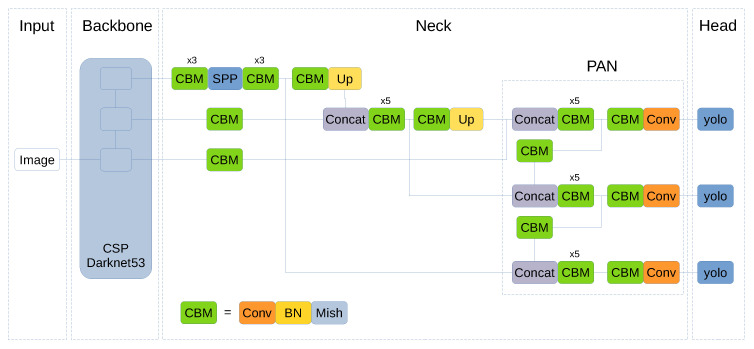
YOLOv4 architecture with all Leaky ReLU functions changed to MISH (Green CBM blocks).

Figure 5 shows YOLOv4 architecture and the changes performed to use MISH activation functions. The CBM block corresponds to Convolution + Batch Normalization + MISH that originally was CBL (namely, Convolution + Batch Normalization + Leaky ReLU). The change for SWISH is done in a similar way resulting in a block called CBS that corresponds to Convolution + Batch Normalization + SWISH.

Table 6 shows results for original YOLOv4 with Leaky ReLU and for SWISH/MISH replacements. It can be seen that the MISH model gets the best average mAP50, followed by SWISH, both leading to better results than the original Leaky ReLU implementation by +1.2% and +0.84% mAP50, respectively. The importance of these results lies in the fact that this enhancement is achieved with a simple yet effective change in the net. However, it comes at a cost: a slight decrease in throughput.

### 3.4. Data Augmentation

The so-called mosaic is one of the improvements added to YOLOv4 [19] for data augmentation. This method merges four training images into one, thus allowing detection of objects outside their normal context. In addition, batch normalization calculates activation statistics from four different images on each layer. This significantly reduces the need for a large mini-batch size.

**Figure 6:**
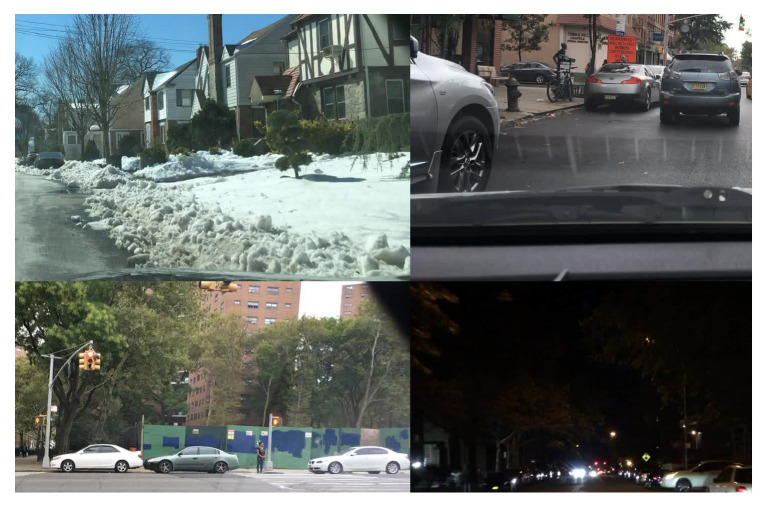
A mosaic example made up of four images.

Figure 6 shows an example of a mosaic augmentation. By using mosaic, we obtained 55.11% mAP50, which surpassed the 52.21% achieved in the experiments with MISH activation functions only.

The combination of mosaic and cutmix did not obtain better results (see Table 7). This may be related to the properties of the dataset and due to the fact that pasting a region from one image into another places objects in a different context. Instead, mosaic combines four different contexts at the same time, which keeps objects in their original context. Data augmentation performance depends on the dataset, and whereas BDD100K does not appear to work better with cutmix, MS-COCO (a generic dataset) shows a good behavior when cutmix is applied to the training process [19].

### 3.5. Grid Size Analysis

YOLOv4 algorithm default configuration uses a 13 × 13 grid that corresponds to an image input of 416 × 416 pixels (grids are regions of 32 × 32 pixels) and YOLOv4-608 corresponds to a 19 × 19 grid. Each grid cell predicts three objects at three different scales (the three YOLO blocks can be seen in Figure 5), which are the original scale grid, 2× and 3× upscaled grids.

In particular, for the 13 × 13 default grid size, the scales are 13 × 13, 26 × 26 and 52 × 52, resulting in a total of 10,647 objects per image. As stated below, each detected object informs about its position, size, objectness, and class confidences $\left(x,y,w,h,c,C_{1},C_{2},\ldots ,C_{n}\right)$. Then, objects are filtered by object confidence *c* and passed to the Non-Maximum Suppression (NMS) algorithm in order to remove redundant detections.

**Figure 7:**
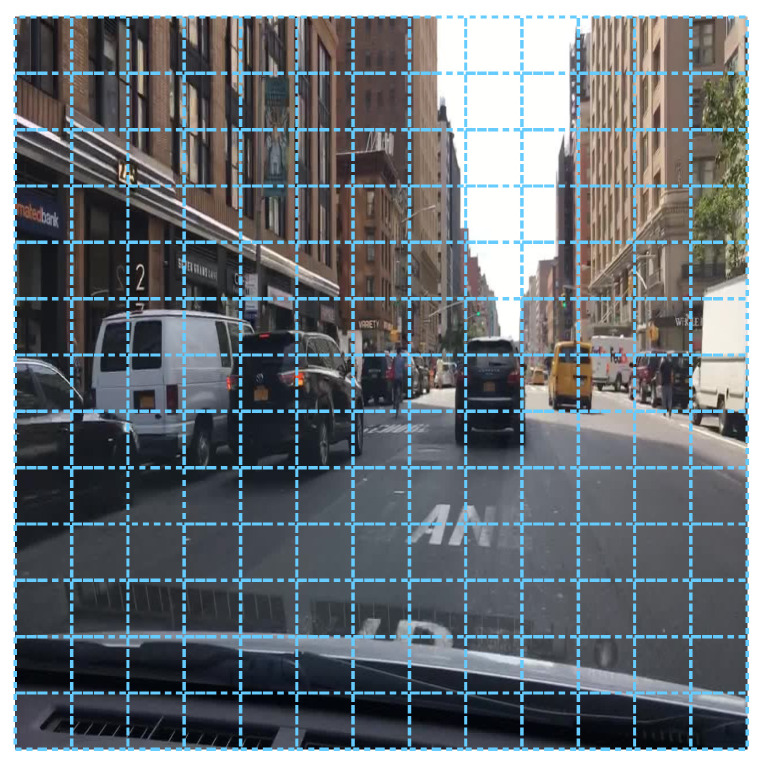
YOLOv4: default 13 × 13 grid.

It could happen that the default 13 × 13 grid (see Figure 7) is not the best setup for the given objects (i.e., the size, density, and distribution of the objects are different for each problem). Therefore, in order to find the optimal size for this problem, an extensive analysis has been carried out for different grid configurations varying width and height from 9 to 32, which correspond to input image sizes from 288 × 288 to 1024 × 1024 pixels. One of the advantages of YOLOv4 architecture is that image input (grid) size for inference can be different from the input size used to train the network, thus allowing a fine-tuning step to get the most from the model at different trade-off points with no need for retraining.

**Figure 8:**
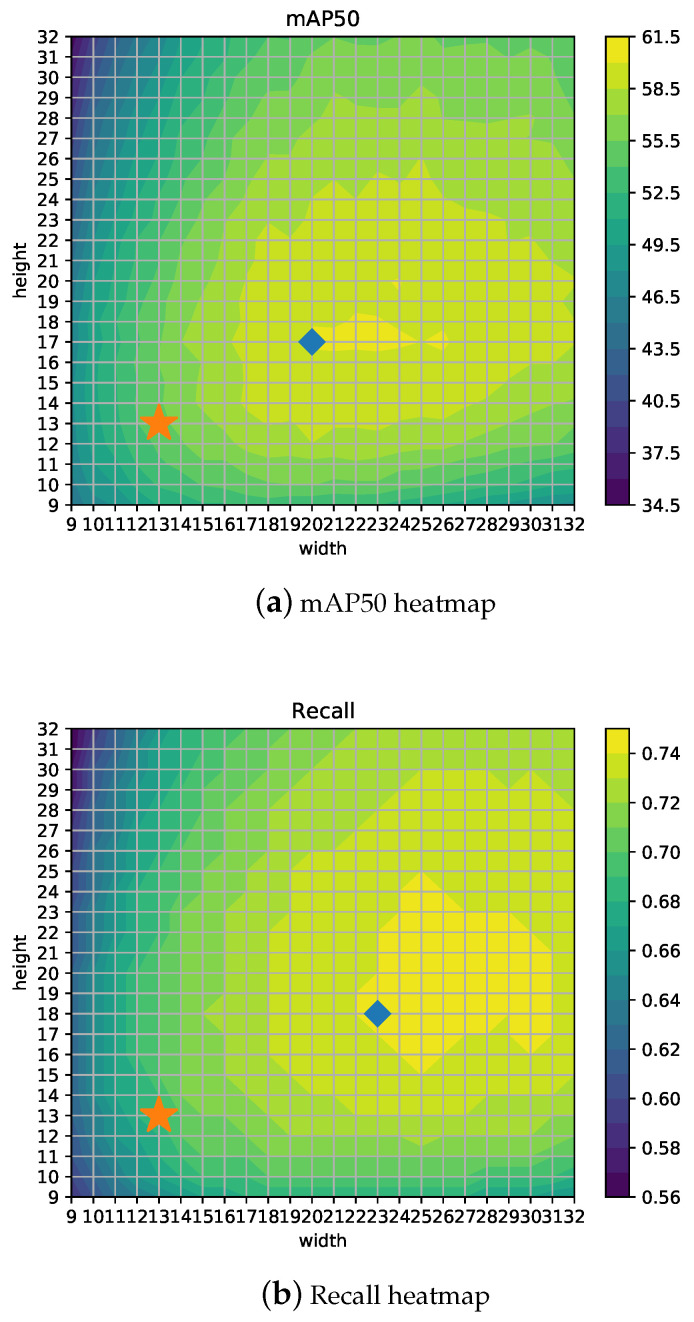

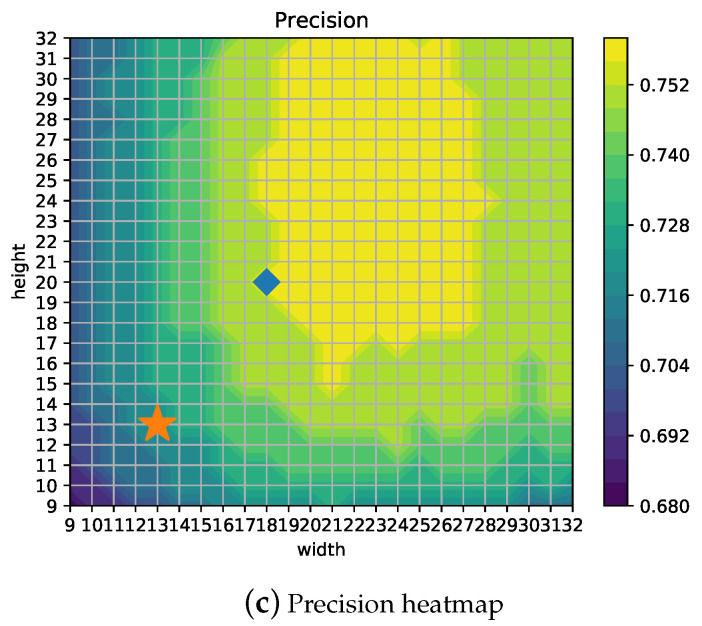
YOLOv4: heatmaps for mAP50, Recall and Precision for different width and height grid configurations.

Figure 8a shows the detector mAP50 performance depending on different configurations for width (*w*) and height (*h*). The best result (marked with a diamond) is obtained for the 20 × 17 configuration getting 60.38% mAP50, which clearly outperforms the 55.11% from the 13 × 13 YOLOv4-416 configuration (indicated with a star). It can be observed that the maximum value is placed along *h* = 17 and also the fact that mAP50 is not symmetrical with respect to the diagonal. Therefore, the results for *w* = 17 and *h* = 13 are better than its symmetric configuration, namely *w* = 13 and *h* = 17, and mAP50 is better for configurations where *w* > *h* in general.

**Figure 9:**
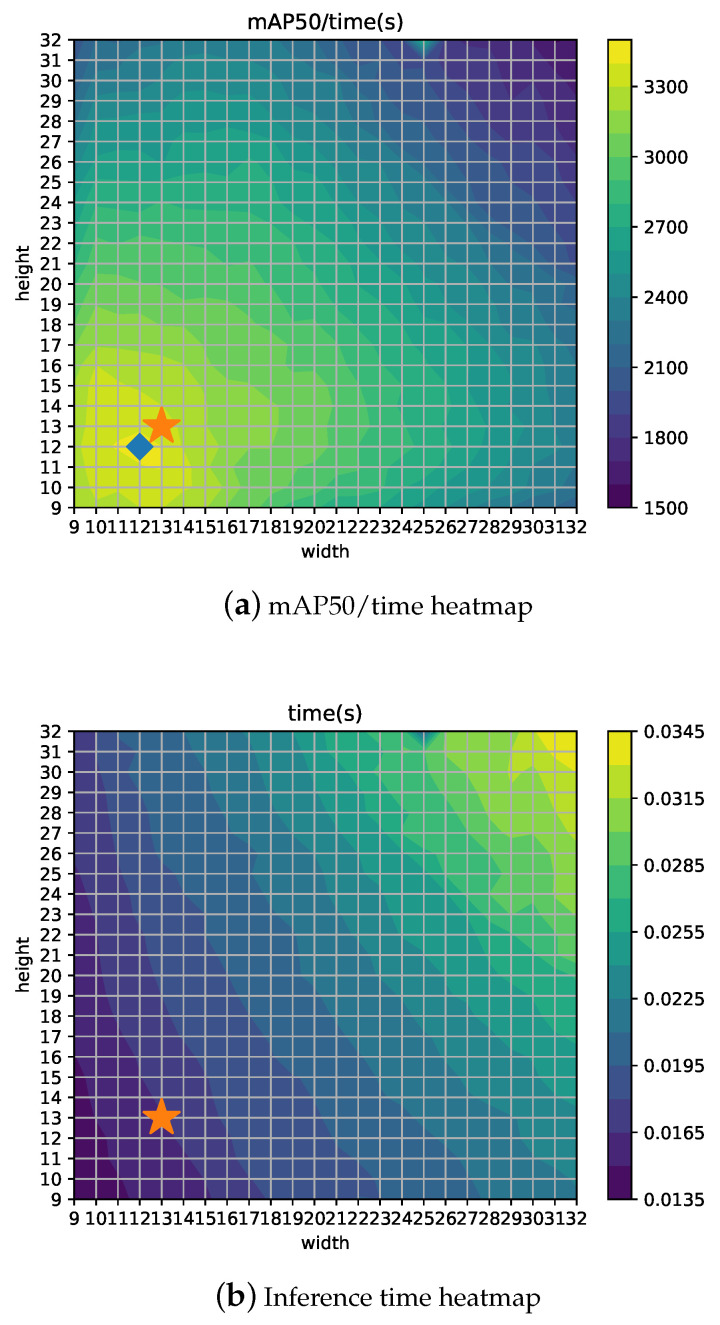
YOLOv4: heatmaps for inference time and mAP50/time ratio for different width and height grid configurations.

Figure 8b,c show heatmaps for Recall and Precision, using the star symbol for the 13 × 13 configuration and a diamond for the best configuration. Depending on the measure to be optimized, the best grid configuration can be different. For example, if Recall is the most important concern for the particular application, a 23 × 18 setup would yield the best results. Thus, in a similar way, if time is a hard constraint, then other configurations such as 12 × 12 would represent the optimal point for mAP/time ratio, as shown in Figure 9b. Moreover, Figure 9a shows the inference time, which is proportional to the grid size as expected (*w* × *h* presents the highest values for the highest *w* and *h*).

Finally, Table 7 shows results for the default configuration (Leaky ReLU) and for the models when replacing Leaky ReLU with MISH or SWISH, applying mosaic and the best grid configuration. It can be noticed that the combination of MISH, mosaic, or cutmix and the 20 × 17 grid offers the best results.

## 4. Conclusions

In this paper, some specific trainings were developed using the BDD100K dataset in order to obtain more precise models to confidently detect the on-road class set in the PRYSTINE project context. First, we trained YOLOv3-416 for three class subsets, with a remarkable increase in detection performance when compared to the original weights from the author with no additional costs. Then, we trained YOLOv4-416 for the 7-class subset, with a moderate improvement in detection performance with respect to YOLOv3-416 but at a cost: a slight decrease in throughput.

Then, we trained YOLOv4-416 for the same 7-class subset but replaced all the original Leaky ReLU activation functions for the YOLOv4 convolutional layers with the new MISH and SWISH functions, and better results were achieved, with slight detection quality improvements from the original Leaky ReLU version. MISH was the function giving the best results.

Additional work was performed by testing mosaic data augmentation, obtaining good results. Finally, the influence of grid configuration for a wide range of values was studied, finding that the best configuration gets a major increase in mAP but at the expense of an important rise in processing time. According to this information, depending on the particular application, it would be possible to select the best working point in each case.

As a remarkable conclusion, we can see that the greater specialization of the networks with a low number of categories generally yields higher precision values for the common classes. Therefore, it is highly advisable to use a network specifically dedicated to the detection of the class group of interest only (and trained with them only as well). The numeric figures presented along the paper can be visually assessed in the analyzed images, where some examples from the BDD100K test subset are passed to the trained nets presented.

## Figures and Tables

**Table 1:** Assignment among the MS-COCO and the BDD100K classes.

BDD100K	Equivalent	Equivalent
Class Name	MS-COCO Class Name	MS-COCO Class Index
Person + Rider	Person	0
Bike	Bicycle	1
Car	Car	2
Motor	Motorbike	3
Bus	Bus	5
Train	Train	6
Truck	Truck	7
Traffic light	Traffic light	9
Traffic sign	-	-

**Table 2:** AP50 for each class with each YOLOv3 model trained with BDD100K: 10-class, 7-class and VRU-class models, when applied to the BDD100K test subset (best result in bold).

BDD100K	10-Class	7-Class	VRU-Class
Class Name	Training	Training	Training
Person	46.67%	47.63%	**48.33%**
Bike	37.73%	40.44%	**41.16%**
Car	68.43%	**68.61%**	-
Motor	34.71%	**38.57%**	36.77%
Rider	40.38%	37.93%	**42.13%**
Bus	58.13%	**58.13%**	-
Truck	**58.61%**	57.67%	-
Train	**0.14%**	-	-
Traffic light	**45.04%**	-	-
Traffic sign	**61.69%**	-	-
**Average**	45.15%	49.85%	42.10%

**Table 3:** AP50 for each class with the original MS-COCO-trained YOLOv3 model from the author when applied to the BDD100K test subset.

MS-COCO Class Name	80-Class Original MS-COCO Training
Person	38.54%
Bicycle	26.68%
Car	51.74%
Motorbike	21.85%
Bus	32.99%
Train	0.54%
Truck	25.83%
Traffic light	15.01%
Traffic sign	-
Average 10-class BDD100K	26.65%
Average 7-class BDD100K	32.94%
Average VRU-class BDD100K	29.02%

**Table 4:** AP50 for each class with YOLOv3 and YOLOv4 models trained with BDD100K when applied to the BDD100K test subset.

BDD100K	YOLOv3-416	YOLOv4-416
Class Name	7-Class Training	7-Class Training
Person	47.63%	**50.32%**
Bike	40.44%	**43.02%**
Car	68.61%	**71.40%**
Motor	38.57%	**39.57%**
Rider	37.93%	**39.80%**
Bus	58.13%	**60.00%**
Truck	57.67%	**61.39%**
**Average**	49.85%	**52.21%**

**Table 5:** Detection quality (mAP50) and throughput (FPS) with YOLOv3 and YOLOv4 models trained with BDD100K when applied to the BDD100K test subset (run on the Nvidia Drive PX2).

Algorithm	BDD100K mAP50 (7-Class)	FPS
YOLOv3-416	49.85%	**14.28**
YOLOv4-416	**52.21%**	11.49

**Table 6:** AP50 for each class with YOLOv4 different activation functions trained with BDD100K when applied to the BDD100K test subset.

BDD100K	Original	MISH	SWISH
Class Name	Leaky Relu YOLOv4	YOLOv4	YOLOv4
Person	50.32%	51.76%	**52.15%**
Bike	43.02%	44.23%	**44.89%**
Car	71.40%	**72.14%**	71.77%
Motor	39.57%	**43.26%**	40.27%
Rider	39.80%	**42.42%**	41.29%
Bus	**60.00%**	59.99%	59.87%
Truck	**61.39%**	60.03%	61.10%
**Average**	52.21%	**53.41%**	53.05%

**Table 7:** YOLOv4 AP50 for each class with different activation functions, data augmentation techniques, and grid size configurations (BDD100K trained and applied to the BDD100K test subset).

Leaky ReLU	✓					
MISH		✓		✓	✓	✓
SWISH			✓			
mosaic				✓	✓	✓
cutmix				✓		
13 × 13 grid (std)	✓	✓	✓	✓	✓	
20 × 17 grid (best)						✓
Person	50.32%	51.76%	52.15%	52.46%	54.15%	**64.47**%
Bike	43.02%	44.23%	44.89%	42.22%	47.38%	**52.54**%
Car	71.40%	72.14%	71.77%	72.85%	73.32%	**79.86**%
Motor	39.57%	43.26%	40.27%	40.48%	42.33%	**47.25**%
Rider	39.80%	42.42%	41.29%	40.58%	44.37%	**49.64**%
Bus	60.00%	59.99%	59.87%	60.89%	62.02%	**64.25**%
Truck	61.39%	60.03%	61.10%	61.41%	62.21%	**64.66**%
**Average**	52.21%	53.41%	53.05%	52.98%	55.11%	**60.38**%

## Abbreviations

The following abbreviations are used in this manuscript:

CNN	Convolutional Neural Network
GPU	Graphics Processing Unit
IoU	Intersection Over Union
mAP50	Mean Average Precision at IoU = 0.5
NMS	Non-Maximum Suppression
MS-COCO	Microsoft Common Objects in Context
SAE	Society of Automotive Engineers
VRU	Vulnerable Road Users
